# Novel LDLR Variant in Familial Hypercholesterolemia: NGS-Based Identification, In Silico Characterization, and Pharmacogenetic Insights

**DOI:** 10.3390/life13071542

**Published:** 2023-07-11

**Authors:** Mohammad Athar, Mawaddah Toonsi, Zainularifeen Abduljaleel, Abdellatif Bouazzaoui, Neda M. Bogari, Anas Dannoun, Faisal A. Al-Allaf

**Affiliations:** 1 Department of Medical Genetics, Faculty of Medicine, Umm Al-Qura University, Makkah 21955, Saudi Arabia; 2 Science and Technology Unit, Umm Al-Qura University, Makkah 21955, Saudi Arabia; 3 Department of Pediatrics, Umm Al-Qura University, Makkah 21955, Saudi Arabia

**Keywords:** familial hypercholesterolemia, cholesterol, low-density lipoprotein receptor, next-generation sequencing, frameshift variant, cardiovascular diseases

## Abstract

Background: Familial Hypercholesterolemia (FH) is a hereditary condition that causes a rise in blood cholesterol throughout a person’s life. FH can result in myocardial infarction and even sudden death if not treated. FH is thought to be caused mainly by variants in the gene for the low-density lipoprotein receptor (LDLR). This study aimed to investigate the genetic variants in FH patients, verify their pathogenicity, and comprehend the relationships between genotype and phenotype. Also, review studies assessed the relationship between the LDLR null variants and the reaction to lipid-lowering therapy. Methods: The study utilised high-throughput next-generation sequencing for genetic screening of FH-associated genes and capillary sequencing for cascade screening. Furthermore, bioinformatic analysis was employed to describe the pathogenic effects of the revealed novel variant on the structural features of the corresponding RNA molecule. Results: We studied the clinical signs of hypercholesterolemia in a Saudi family with three generations of FH. We discovered a novel frameshift variant (c.666_670dup, p.(Asp224Alafs*43) in the LDLR and a known single nucleotide variant (c.9835A > G, p.(Ser3279Gly) in the APOB gene. It is thought that the LDLR variant causes a protein to be prematurely truncated, likely through nonsense-mediated protein decay. The LDLR variant is strongly predicted to be pathogenic in accordance with ACMG guidelines and co-segregated with the FH clinical characteristics of the family. This LDLR variant exhibited severe clinical FH phenotypes and was restricted to the LDLR protein’s ligand-binding domain. According to computational functional characterization, this LDLR variant was predicted to change the free energy dynamics of the RNA molecule, thereby affecting its stability. This frameshift variant is thought to eliminate important functional domains in LDLR that are required for receptor recycling and LDL particle binding. We provide insight into how FH patients with a null variant in the LDLR gene respond to lipid-lowering therapy. Conclusions: The findings expand the range of FH variants and assist coronary artery disease preventive efforts by improving diagnosis, understanding the genotype-phenotype relationship, prognosis, and personalised therapy for patients with FH.

## 1. Introduction

Familial Hypercholesterolemia is a prevalent genetically inherited disease characterised by higher cholesterol levels bound to LDL that can accumulate in the eyes, tendons, blood vessels, and skin, forming a typical picture of the disease [1]. Thus, FH can lead to severe cardiovascular events and sudden death [2]. Although these lethal sequelae could be prevented with early diagnosis and management, many patients are underdiagnosed, undertreated, or even untreated [3,4,5]. The epidemiology of this manageable, life-threatening disease in Saudi Arabia is not yet well known. Recent studies from previously investigated populations appear to suggest that the actual occurrence of FH is far higher than reported. They have shown a considerable variation in the distribution of the disease among different countries [6].

The type and nature of the causative variants are unknown, and the disease frequency is not yet known. Therefore, knowing the signs and symptoms of the disease is not enough to diagnose it, as patients with a mild form of FH will not show this typical picture. On the other hand, many normal individuals exhibit high levels of LDL-C due to their unhealthy lifestyles. Three well-known criteria were developed to facilitate the diagnosis of such disease, and they are commonly used in clinical practice: the Simon Broome, the Dutch, and the US MEDPED Criteria. In the Simon Broome criteria, patients are considered either definite or possible for FH depending on their clinical features, family history, biochemical tests, and molecular studies [7]. The Dutch criteria is a modification of the Simon Broome criteria; it depends on a scoring system according to which the patients are categorised into four different groups, i.e., definite, probable, possible, and unlikely FH. In the US MEDPED Criteria, patients are diagnosed according to their age, their relatives’ cholesterol levels, and nothing else [8]. By using these criteria, FH can be screened and diagnosed.

Genetically, FH could be inherited either in an Autosomal Dominant (AD) or or autosomal recessive (AR) fashion [9], with the former being the most common type. FH could also be classified as either Homozygous (HoFH), which is the most severe form and could be very difficult to treat, but thankfully is so rare, or Heterozygous (HeFH), the most common type; this form responds very well to treatment. The mean age of developing cardiovascular disease (CVD) is reported to be 20 years in individuals with HoFH, regardless of gender [10]. Nevertheless, there is an apparent difference between males and females among those with HeFH. In contrast to females with HeFH, who normally acquire CVD at the age of 51–52, men with HeFH are likely to encounter CVD at an average age of 42–46 years [11,12]. Variants in any of the genes that encode the LDL receptor (LDLR), apolipoprotein B (APOB), or proprotein convertase subtilisin/kexin type 9 (PCSK9) are causative of the AD type of the disease [13,14]. The LDLR gene accounts for more than 90% of the variants, and about 2300 putative pathogenic variants have been identified [15]. The AR-type of FH is caused by a variant in the LDL receptor adapter protein 1 (LDLRAP1) gene [16]. Additionally, genetic variants in secondary FH genes like APOE, ABCG5/8, and LIPA are also reported to influence the cholesterol level in the blood [17,18].

In recent years, the pharmacological treatment for FH has advanced significantly. Presently, statins, ezetimibe, and PCSK9 inhibitors are the medications of choice for treating FH in the clinic. However, many patients are not identified in time to receive appropriate care, which has been identified as a global health problem [19]. Individual responses to lipid-lowering treatment (LLT) with statins and/or PCSK9 inhibitors have been found to differ significantly in previous studies [20]. Individual variations in response to LLT are important clinical issues to investigate since they might alter cardiovascular outcomes [21]. However, the reason for the disparity in people’s responses to LLT is still unknown. Various medical and genetic factors have been suggested to influence the reaction in the general population [22]. Thus, within FH, genetic differences are likely to impact an individual’s sensitivity to LLT. Dissimilarities in cardiovascular risk related to genetic characteristics in FH might be partially explained if the presence or kind of pathogenic mutations changed the patient’s response. Several investigations have found that the response to statins and PCSK9 inhibitors varies depending on the kind of variation [23]. Nevertheless, contradictory studies have necessitated further exploration to clear up the association between genetic features and responses to statins and PCSK9 inhibitors [24].

The Saudi Arabian population is mainly consanguineous and has a high incidence of genetic disorders [25]. However, in Saudi Arabia, the number of genetic studies conducted to determine the molecular characteristics of FH is still limited [1,2,3,9,25,26,27,28,29,30]. The FH variant spectrum in Saudi Arabia remains to be further elucidated. The consanguineous background of the Saudi population is expected to offer a potential chance to identify novel genetic variants and variant segregation patterns of the FH candidate genes [3,9,26,29]. As a result, the current study was created to identify the genetic and molecular basis of FH in a Saudi family. We initially performed targeted NGS on the close blood relatives of the FH proband. We then determined the variant segregation in the other family members by capillary sequencing, followed by genotype-phenotype correlations. Furthermore, research evaluating the link between LDLR null variants and response to lipid-lowering therapy should be reviewed.

## 2. Material and Method

### 2.1. Study Subjects

This study was part of a comprehensive study focusing on screening FH variants by exome sequencing of targeted FH-associated genes in the Saudi population. Clinical screening, biochemical profiling, targeted Next-generation sequencing, and variant segregation analysis were performed on the FH family living in the western Saudi Arabia region. The sample collection and study were conducted following the Research Ethics Committees’ regulations at Umm Al-Qura University, Makkah, Saudi Arabia, and all subjects gave informed consent. 

### 2.2. Targeted Ion Torrent Next-Generation Sequencing (NGS)

NGS was conducted using samples from the proband’s first-degree blood relatives [mother (I.2), brother (II.1), sister (II.2), and daughter (III.1)]. Following the manufacturer’s guidelines, the QIAamp DNA Blood Mini Kit (Qiagen) was used to extract genomic DNA from EDTA-treated whole blood. The NanoDrop 2000 instrument was used to determine the quantity and purity of extracted DNA (Thermo Fisher Scientific, Waltham, MA, USA). We created an Ion AmpliSeq panel with exons and surrounding intron regions from four FH-related genes (LDLR, APOB, PCSK9, and LDLRAP1). To prepare the library for sequencing on the Ion Chef System, the tailored Ion AmpliSeq panel primers pools and Ion AmpliSeq Kit for Chef DL8 were used (Thermo Fisher Scientific, Waltham, MA, USA). The Ion Chef system was used to prepare emulsion PCR-based templates and load chips, with the Ion PGM Hi-Q View Chef Kit and the Ion 318 Chip Kit v2 BC (Thermo Fisher Scientific, Waltham, MA, USA). Finally, the Ion PGM sequencer was used to conduct the sequencing (Thermo Fisher Scientific, Waltham, MA, USA). 

### 2.3. Analysis of NGS Data

The NGS data analysis was completed in accordance with Athar et al. [31]. In brief, amplicon sequences of the target region of our constructed Ion AmpliSeq panel genes were matched to the human reference genome hg19 (GRCh37) using Torrent Suite software after subsequent DNA sequencing on Ion PGM (version 5.12.0; Thermo Fisher Scientific). The Variation Caller Plugin v5.2 (Thermo Fisher Scientific) was used to generate a variant call format (vcf) file for each sample. To assess the functional repercussions of the variants, we annotated the vcf file with the ANNOVAR program (http://wannovar.wglab.org/, accessed on 7 December 2022). The in silico methods SIFT and PolyPhen-2 were used to assess the pathogenicity of the missense variants [32]. If a variant does not have a rsID and is not listed in the literature or a public database like the Human Gene Mutations Database (HGMD), ClinVar, or LOVD (https://databases.lovd.nl/shared/genes, accessed on 15 March 2023), we will consider it to be novel. Using the CLC Genomics Workbench, we examined the amino acid conservation near the variant position (QIAGEN Bioinformatics, Hilden, Germany).

### 2.4. Validation of Variants and Segregation Analyses

Sanger sequencing was used to confirm the deleterious variants identified by NGS analysis in the LDLR and APOB genes. After the variants were confirmed, other first-degree blood relatives (I.3, II.3, II.5) were examined for co-segregation. The HotStarTaq Plus Kit was used for PCR (Qiagen, Hilden, Germany). PCR amplicons were cleaned up using magnetic beads (Agencourt AMPure kit; Beckman Coulter, Brea, CA, USA). The purified PCR products were sequenced with the BigDye Terminator v3.1 cycle sequencing kit (Applied Biosystems, Foster City, CA, USA). Sanger sequencing was performed on a Genetic analyser called the ABI 3500 (Applied Biosystems, Foster City, CA, USA).

The family’s variant segregation was found through a meticulous examination of each family member’s variant status.

### 2.5. Functional Analysis of LDLR Variant on RNA Secondary Structure

The effects of pathogenic variants on RNA secondary structures can be examined to gain insight into potential functional outcomes. We were using the RNA Fold web server prediction tool (http://rna.tbi.univie.ac.at/cgi-bin/RNAWebSuite/RNAfold.cgi, accessed on 25 January 2023) to predict the best possible secondary structure of an RNA molecule. This tool calculates the backbone traces and the differences in the minimum free energy (MFE) values [33]. This tool requires native and wildtype RNA sequences in FASTA format to calculate the probability of the base-pair matrix, partition feature, and configuration of centroid molecules. The products of RNA folding include an interactive string depiction of the secondary structure of the RNA and a mountain plot depicting the conformational changes of energy variations among native and mutated sequences. MFE differences among native and mutated RNA structures were examined to determine the consequences of disease-causing variation on their secondary structural properties.

## 3. Results

### 3.1. Clinical Characteristics of the FH Family

The proband was diagnosed clinically as homozygous for FH using the Simon Broome criteria [7]. The proband presented severe clinical features of FH, including xanthoma and xanthelasma. He was resistant to statin therapy and was on LDL apheresis. Unfortunately, we could not collect samples from the proband, as he died many years ago in his early twenties due to sudden cardiac death. His parents were first cousins; his father (I.1) died when he was 52. We collected samples from his mother, maternal aunt, sisters, brother, and daughter. Table 1 shows the family’s lipid profile, clinical manifestations, and therapeutic interventions. The proband’s mother (I.2) has undergone angiography and bypass surgery due to severe myocardial infarction. The brother (II.1) also underwent percutaneous coronary intervention due to myocardial infarction. The lipid level of the proband’s mother (I.2), brother (II.1), and daughter (III.1) was high, while Table 1 shows when they were under treatment. The lipid profile levels of the proband’s sisters (II.2 and II.3) were moderate, as shown in Table 1. However, the proband’s other sister (II.6) had an increased LDL cholesterol level (5.63 mmol/L). All the family’s cardiovascular events and increased serum lipid profiles refer to premature atherosclerosis, which is coherent to severe heterozygous FH.

### 3.2. Variants Identification in the FH Family

**Figure 1:**
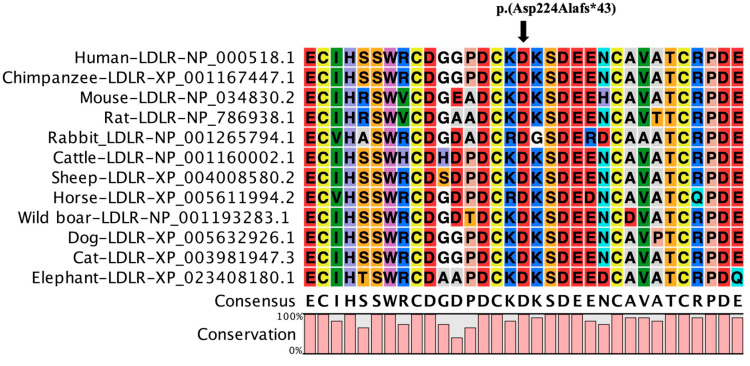

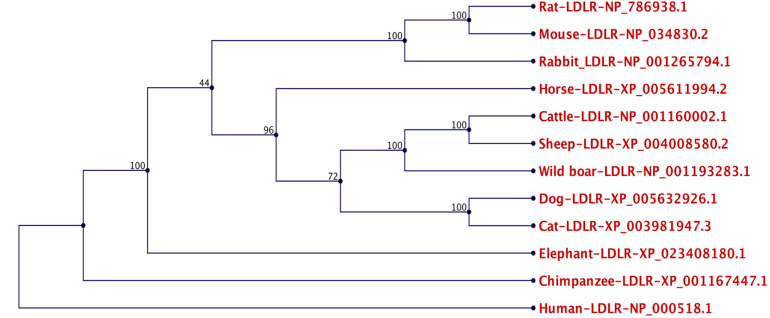
Protein alignment of the LDLR ligand-binding domain sequence across different mammalian species shows that the regions around the variant are highly conserved. The position of the variant is marked by a black arrow.

Next-generation sequencing was performed on samples from the proband’s first-degree blood relatives [mother (I.2), brother (II.1), sister (II.2), and daughter (III.1)]. NGS analysis for this Saudi family that lives in the western region of Saudi Arabia showed two different variants—a novel frameshift variant in exon 4 of the LDLR gene (c.666_670dup, p.(Asp224Alafs*43) and a known missense variant in exon 26 of the APOB gene (c.9835A > G, p. (Ser3279Gly) (Table 2). This duplication variant c.666_670dup produces a frameshift in the LDLR gene’s coding sequence and then replaces the native amino acid aspartic acid with variant alanine at the 224th position, preceded by 43 nonsense residues, eventually leading to a premature stop gain signal that truncates the LDLR protein at the 267th amino acid (Asp224Alafs*43). The said LDLR variant is thought to produce a prematurely truncated protein that is likely to decay via nonsense-mediated protein decay. This frameshift variant is found in the LDLR protein’s ligand-binding domain. Moreover, the above frameshift variant was found to be situated in a phylogenetically highly conserved region of a protein sequence along all multiple species such as *Chimpanzee*, *Mouse*, *Rat*, *Rabbit*, *Cattle*, *Sheep*, *Horse*, *Wild boar*, *Dog*, *Cat*, and *Elephant* (Figure 1). Therefore, according to the standards and ACMG guidelines [15], the LDLR variant p.(Asp224Alafs*43) is strongly predicted to be pathogenic. Computational prediction methods like SIFT and Polyphen-2 have indicated the APOB variant p. (Ser3279Gly) as deleterious (Table 2). This APOB variant can be considered a rare variant (MAF:0.0044) as reported in the Exome Aggregation Consortium (ExAC) database. This missense variant p. (Ser3279Gly) is found in the APOB protein’s LDLR binding domain.

### 3.3. Variants Segregation Analysis

**Figure 2:**
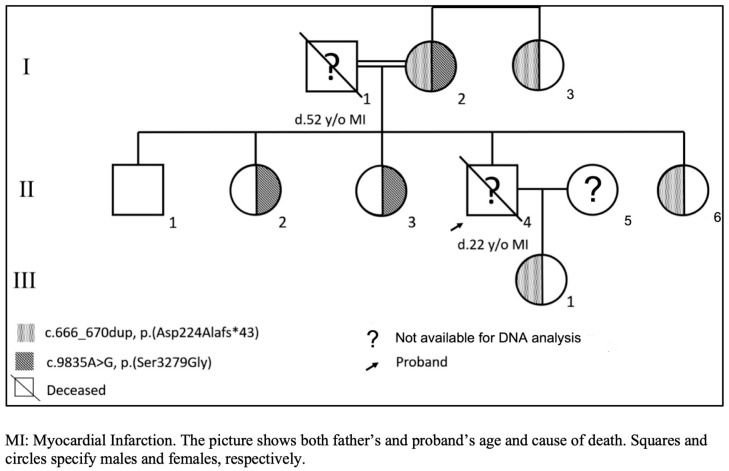
Pedigree of a Saudi family with familial hypercholesterolemia.

**Figure 3:**
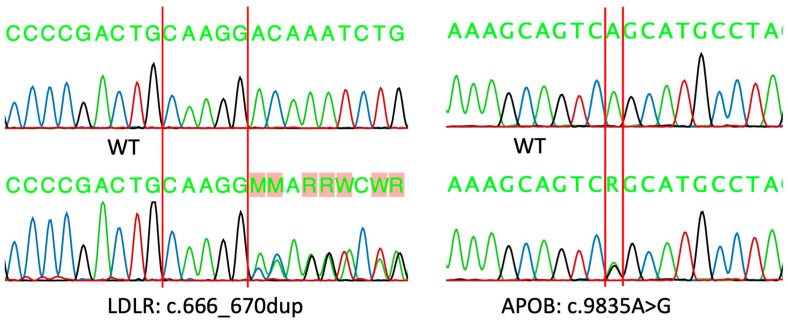
Representative DNA sequence from the family control (Wild type; WT) and heterozygous LDLR and APOB variants of the studied family.

The autosomal dominant mode of inheritance of variants in this FH family has been confirmed by capillary sequencing results. Figure 2 demonstrates the pedigree of the FH family. The family’s variant dispersion is as follows; the mother of the proband was found to be double heterozygous, carrying both variants. The daughter (III.1), one sister (II.6) and maternal aunt (I.3) were found to be heterozygous for LDLR variant p.(Asp224Alafs*43). Moreover, two sisters (II-2 and II.3) were heterozygous for APOB variant p.(Ser3279Gly). Although the brother’s history (I.1) was so suggestive, we could not identify any variant in the common FH-associated genes (LDLR, APOB, PCSK9, and LDLRAP1). Figure 3 demonstrates both variants on exon 4 of the LDLR gene and exon 26 of the APOB gene. Dyslipidemia correlates with the segregation arrangement of the frameshift LDLR variant (Table 1).

### 3.4. Functional Significance of LDLR Pathogenic Variant on RNA Structure

**Figure 4:**
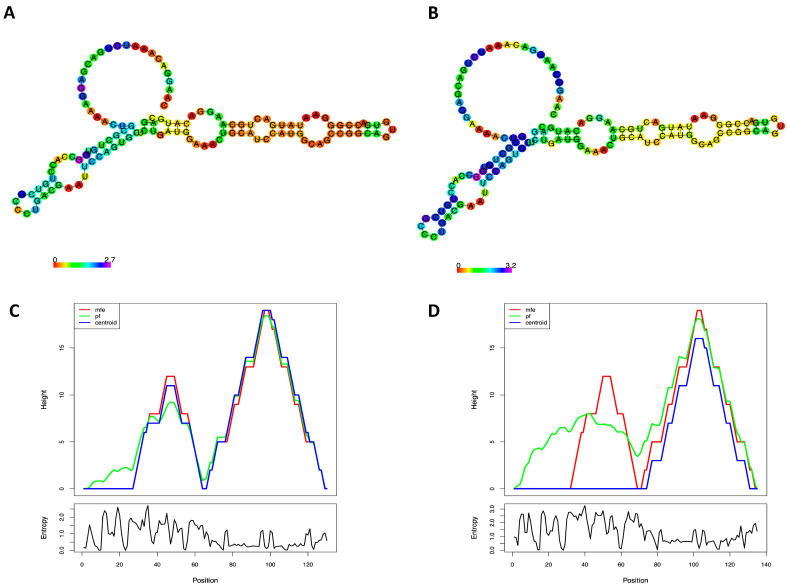
Human LDLR RNA secondary structure estimated by RNA Fold. Predictions of RNA secondary structure for wildtype (**A**) and mutant c.666_670dup (**B**) using the minimum free energy (MFE) assessments of nucleotide base pairing, as depicted by the colour gradient on a scale of 0–3. Mountain plot (MP) depiction of MFE, thermodynamic ensemble (pf), and centroid structure predictions of the LDLR native (**C**) and mutant c.666_670dup (**D**) RNA secondary structures. MP depicts secondary structures in height vs position plot, with helices in slopes, loops in plateaus, and hairpin loops in peaks. The bottom graph illustrates the entropy of the predicted RNA structure, with higher entropy indicating lower stability.

To evaluate stability, RNA secondary structure can be predicted using free energy minimisation with nearest neighbour parameters [34]. LDLR mutant mRNA molecules (c.666_670dup) have significantly lower secondary structure stability with −14.40 kcal/mol than native LDLR mRNA molecules (Minimum free energy, MEF was −26.40 kcal/mol), according to the estimate of the MFE of LDLR centroid structures. Accordingly, it is anticipated that the reduced mRNA stability caused by c.666_670dup will impact the mRNA folding configuration and the creation of tertiary structures (Figure 4).

## 4. Discussion

The most frequent cause of FH is variants in the LDLR gene that reduce the hepatic elimination of LDL from the bloodstream [29]. Over 2300 genomic variants in LDLR have been identified [15]. This article describes a novel LDLR frameshift and a known APOB missense variant discovered in a Saudi family living in western Saudi Arabia. Thirty-three percent of LDLR variants contain frameshift, nonsense, and large genomic rearrangements, all deemed null variants and, as a result, pathogenic. It has been assumed that all variants that produce a null allele do not require functional proof to be a cause of the disease due to their harmful effect on the LDLR protein. Missense variants, on the other hand, need functional corroboration to be characterised as disease-causing or benign [35]. When analysing the distribution of LDLR variants, it is notable that LDLR exon 4, the longest exon, has the highest number of described variants, including a significant number of functionally characterised pathogenic variants [35]. We discovered a novel frameshift variant in the LDLR gene’s exon 4, which is regarded as a functionally deleterious variant and is correlated to the previous findings, as stated above. Additionally, the truncated transcript of the frameshift variant p.(Asp224Alafs*43) raises the likelihood of hypercholesterolemia. Table 1 shows a correlation between the clinical profile and the frameshift variant’s segregation arrangement. The proband’s two siblings (II.2 and II.3) were found to be negative for the LDLR variant, which corresponds with a low cholesterol level in the blood (Table 1). 

Understanding structure-function correlations and developing therapies and diagnoses that target RNA depend on knowing the structure of the RNA molecule. Premature termination codons (PTC) in mRNAs have the potential to activate nonsense-mediated mRNA decay (NMD) [36] or leave a noticeably truncated LDLR protein that would subsequently be degraded by the ubiquitin-mediated proteasomal pathway [37]. Both mRNA quantity and stability were observed to be decreased in human mRNAs with PTC [38]. Our analysis of RNA stability indicates that the LDLR, c.666_670dup, is anticipated to destabilise the mRNA structure and eventually disrupt its folding pattern. Translation of the allele containing the frameshift variant p.(Asp224Alafs*43) truncates the proteins by 265 amino acids and is considered a receptor-negative variant. Consequently, we predict that the variant p.(Asp224Alafs*43) refers to the class 1 group of LDLR variants because it is a loss-of-function (LoF) variant or a null allele that prevents the synthesis of LDLR protein [39].

In silico prediction tools (SIFT and PolyPhen 2) identified the APOB variant p. (Ser3279Gly) as harmful. The molecular docking study also demonstrated that the APOB variant might impact binding affinity [28]. It is unlikely that the clinical presentation in this family is unrelated to the APOB variant, as evidenced by cascade screening and segregation analysis. However, compared to patients with only the LDLR gene variant, patients with both LDLR and APOB variants had no additional impact on FH clinical manifestations (Table 1).

This participant (II.1) tested negative for the family variant despite having a high total cholesterol level (Figure 2). As a result, this participant could have variations in other genes. However, population studies show that 15-40% of people are negative for pathogenic variants in the three principal FH genes (LDLR, APOB, and PCSK9). Nonetheless, they have a clinical manifestation of FH (non-FH) that may be linked to polymorphisms in several other genes, including Apolipoprotein E (APOE), signal transducing adaptor family member 1 (STAP1) and ATP-binding cassette subfamily G members 5 and 8 (ABCG5/8) [29]. Because this family has a high rate of consanguinity, the proband’s father could have the same LDLR variant. The proband was diagnosed clinically homozygous, and early demise suggested that both paternal and maternal alleles might be affected by the same LDLR variant.

The majority of FH patients continue to receive diagnoses into their later years and receive inadequate treatment [29]. Additionally, a cross-sectional study by Batais et al. [40] showed a substantial lack of awareness, practicing, and diagnosis of FH among Saudi Arabian health professionals. Because the disease does not show symptoms at first, some people do not realise they have it until they have a myocardial infarction, usually between the ages of 40 and 50. The disease is dangerous at this point and can cause sudden death or other cardiovascular incidents. American and European recommendations state that FH sufferers should be detected as early as possible so that LDL-cholesterol-lowering therapy can begin in the early years of life to strengthen the patient’s outcome [41]. It is essential to the prevention of coronary heart disease to identify high-risk individuals through cascade screening of families, who are not receiving treatment, have an LDL cholesterol level greater than five mmol/L, and should be referred to a specialist. A diagnosis must be made at a young age to ascertain and monitor LDL cholesterol levels [41]. Additionally, the cumulative burden of LDL cholesterol can be reduced when lipid-lowering medications are started early [1,41]. Therefore, this study’s main objective was to find genetic variations in FH patients and their first-degree relatives, particularly children, in order to understand the molecular basis of FH in Saudi Arabia.

The proband’s xanthoma and xanthelasma were severe clinical signs of FH. He was resistant to statin therapy and was on LDL apheresis. In contrast, the proband’s daughter only had hypercholesterolemia and lacked other clinical characteristics. According to Fraser et al., lipid age was an indicator for predicting arteriosclerosis-related cardiovascular disease risk [42]. The phenotype of the FH patient should be contemplated considering age. Additionally, the proband’s daughter may experience fewer cardiovascular events if long-term medical care and lipid-lowering medications are started early.

Additionally, we review studies that assessed the relationship between the LDLR null variants and the reaction to lipid-lowering therapy (Table 3). Variants in the LDLR gene, which increase susceptibility to FH and coronary artery disease, might selectively alter the therapeutic effect of anti-lipid treatment. As such, pharmacogenetic analysis primarily concentrates on identifying LDLR variants, as examined in Table 3, that show the pharmacogenomic variations associated with lipid-lowering therapy response in FH patients with LDLR null/defective variants. People having a null variant (frameshift/nonsense) were found to have decreased responses with increased cholesterol compared to those carrying a defective (non-frameshift) or no mutation [43,44,45,46]. Furthermore, FH patients with a null mutation in the LDLR gene had a higher prevalence of CVD than those with a defective version. Despite being at high risk of CVD and on aggressive anti-lipid regimens, most of these people did not reach the therapeutic LDL-C targets [47,48]. In contrast, Vohl et al. reported that the proportion of patients who met LDL-C targets was higher in null mutants than in impaired ones [47]. These findings suggest that modifying the LDLR should be a potential pharmaceutical goal for treating FH. Many non-statin medications, including ezetimibe, PCSK9 inhibitors, mipomersen, and lomitapide, efficiently regulate cholesterol levels and might be prescribed as monotherapy or combination therapy in FH patients [44,49,50,51,52,53]. For resistant or non-adherent statin-induced muscular discomfort, the most recent guidelines recommend escalating therapy with non-statin medicines in addition to maximum statins [54]. Many biogenetic analyses have been undertaken to investigate these characteristics, as shown in Table 3. Nonetheless, further pharmacogenomic probes are required to fully comprehend the medical response in the FH population.

Patients with FH have varying responses to treatment. Among people with FH, the range of LDLR variations can affect subjects’ traits and responses to therapy to reduce cholesterol levels. It has been reported that people with null variants show higher LDL levels and increased chances of early coronary heart disease (CHD) than those with defective carriers; additionally, null variants were observed to not respond well to statin drugs [55]. Moreover, the number of alleles present was seen to impact treatment effectiveness; for instance, patients suffering from heterozygous and homozygous FH who were given PCSK9 inhibitors saw a decrease in LDL-C levels of 56% and 38%, respectively [56]. Another study concerning responses to PCSK9 inhibitor therapy concluded that there was no distinction in responses between different groups of heterozygous FH individuals. Remarkably, reactions to PCSK9 inhibitor treatments were significantly less successful for carriers of compound heterozygous or homozygous variants [57]. In the FH family studied here, statins managed to some extent to regulate LDL-C levels. However, PCSK9 inhibitors are recommended in addition to statins for optimal LDL-C levels as they have proven more effective in heterozygous patients [55]. Consequently, analysing and categorising LDLR variants can be used to pick more personalised lipid-lowering therapies and dyslipidemia management plans, eventually resulting in improved patient results.

The current study was constrained in some ways. First, the study only included seven members of one family. More patients need to be studied to comprehend the full range of variants that cause FH in Saudi Arabia. Second, while co-segregation analyses are essential for determining a variant’s pathogenicity, in vitro functional analyses are required to support or refute the pathogenic potential of an identified variant. Lastly, most of the studies focused on LDLR gene exon variants. Variants in the intron regions of LDLR have been shown to influence the splicing of mRNA precursors and cause FH [58]. The LDLR intron region variation mechanism and its impact on the onset and progression of FH should be investigated in subsequent research.

## 5. Conclusions

We discovered a novel frameshift variant of LDLR that appears to be involved in the pathogenesis of FH. Extending the spectrum of LDLR variants and investigating genotype-phenotype correlation is critical for understanding the pathogenesis of FH. This loss-of-function variant p.(Asp224Alafs*43) was expected to change the RNA molecule’s free energy dynamics, affecting its stability, and was presumed to eliminate key LDLR functional domains and subsequently degrade. Also, provide insight into the pharmacogenetic variation linked to LLT response in FH patients with a null/defective LDLR variant. These people may benefit from the use of newly approved lipid-lowering medications. Genetically directed lipid-lowering treatment boosts total safety, advances drug compliance, and enables long-term therapy. This disorder is anticipated to be detected early and treated long-term, thereby reducing cardiovascular disease, and increasing life expectancy, particularly in young people.

## Figures and Tables

**Table 1:** Description, lipid profile, clinical history, and treatment of the studied family members with the novel LDLR and known APOB gene variants.

Family Members	Mother (I-2)	Maternal Aunty (I-3)	Brother (II-1)	Sister (II-2)	Sister (II-3)	Sister (II-6)	Daughter (III-1)
Sex	Female	Female	Male	Female	Female	Female	Female
Age (Years)	71	68	46	44	39	31	12
Total Cholesterol (mmol/L)	6.4	14.06	7.8	5.67	4.34	6.95	8.9
LDL-C (mmol/L)	4.39	4.4	2.54	4.02	2.9	5.63	8.1
HDL-C (mmol/L)	0.93	0.72	1	1.14	1.65	1.2	1.4
Triglycerides (mmol/L)	2.37	11.51	9.29	2.51	1.08	0.88	1.6
History	MI, angiography and bypass surgery	Open heart surgery	MI, and PCI	-	-	-	-
Treatments	Rosuvastatin Omega-3	Rosuvastatin Fenofibrate	Rosuvastatin Ezetimibe	-	-	-	Simvastatin Omega-3
Family History of CAD	Positive	Positive	Positive	Positive	Positive	Positive	Positive
LDLR variant	p.(Asp224Alafs*43)	p.(Asp224Alafs*43)	-	-	-	p.(Asp224Alafs*43)	p.(Asp224Alafs*43)
APOB variant	p.(Ser3279Gly)	-	-	p.(Ser3279Gly)	p.(Ser3279Gly)	-	-

Lipid profile results are with treatment in the mother, brother, and daughter of the proband. LDL-C, low-density lipoprotein cholesterol; HDL-C, high-density lipoprotein cholesterol; CAD, coronary artery disease; MI, myocardial infarction; PCI, Percutaneous coronary intervention.

**Table 2:** Characterisation of *LDLR* and *APOB* genetic variants identified by NGS and their pathogenicity analysis.

Gene	Genetic Variant	dbsnp	Exon	Status	Pathogenicity Analysis				
					Functional Domain	SIFT	PolyPhen-2	Allele Frequency (ExAC)	ACMG Classification
LDLR	c.666_670dup, p.(Asp224Alafs*43)	Frameshift	4	Novel	Ligand-binding	N/A	N/A	-	Pathogenic
APOB	c.9835A > G, p.(Ser3279Gly)	Missense	26	rs12720854	LDLR binding	Damaging	Probably Damaging	0.0044	Variant of uncertain significance

N/A: not applicable.

**Table 3:** Pharmacogenetic variability in response to lipid-lowering therapy in FH patients with LDLR null/defective variants.

Significant LDLR Variant	Patients	Population	Sample Size	Treatment and Daily Dose	Clinical Outcomes	References
Null and defective	Het-FH	British	109	Simvastatin	Patients with null variants have less LDL-C reduction than those with defective variants.	Heath et al., 1999 [45]
Null and defective	FH	Spanish	55	Simvastatin 20 mg	Patients with defective rather than null variants are more likely to have low HDL-C and poor statin response.	Chaves et al., 2001 [46]
Null and defective	Het-FH	Canadian	63	Atorvastatin 20 mg	LDL-C reduction is higher in patients with null than defective variants.	Vohl et al., 2002 [47]
Null and defective	Het-FH	Spanish	811	Simvastatin or atorvastatin 80 mg ± bile acid sequestrant	Patients with null rather than defective variants have a higher risk of CVD and TC.	Alonso et al., 2008 [11]
Null and defective	FH	Spanish	387	Maximum statin doses ^#^ + ezetimibe 10 mg	Patients with null rather than defective variants have a poor LLT response and a higher risk of CVD.	Mata et al., 2011 [48]
Defective and negative	Hom-FH	South African	8	Evolocumab 140–420 mg every 2–4 weeks for 3 months	Evolocumab decreases LDL-C in LDLR-deficient patients but not in negative cases.	Stein et al., 2013 [49]
Null and defective	Het-FH	Brazilian	156	Atorvastatin 10, 20 or 40 mg	Patients with a defective variant have a more significant reduction in LDL-C than those with a null variant.	Santos et al., 2014 [35]
Defective and negative	Hom-FH	Multiple countries	50	Evolocumab 420 mg every 4 weeks for 3 months	The response to evolocumab is LDLR-genotype dependent, with increased sensitivity in LDLR-defective individuals.	Raal et al., 2015 [50]
Null and defective	FH	Spanish	4132	Maximum statin doses ^#^ + ezetimibe 10 mg	Null variants had a greater incidence of CVD and a poorer LLT response than defective variants.	Perez de Isla et al., 2016 [23]
Defective and negative	Hom-FH	South African	22	Mivastatin and evolocumab	Defective LDLR variants are responsive to evolocumab, whereas negative LDLR variants are not.	Thedrez et al., 2018 [51]
Null variant in both alleles	Hom-FH	Multiple countries	69	Atorvastatin 80 mg, ezetimibe 10 mg, lomitapide, and alirocumab 150 mg/2 weeks for 12 weeks	Lipid profile management is improved with alirocumab.	Blom et al., 2020 [52]
p.(D445*)	Hom-FH	Saudi	2	Ezetimibe, Simvastain	Statin resistant and managed with LDL-apheresis.	Al-Allaf et al., 2014 [26]
Stop Gain Variants (p.C231* and p.R744*)	Het-FH (2 families)	Saudi	3	Atorvastatin 40 mg	The high cholesterol profile was managed appropriately after increasing the Atorvastatin dosage to 40 mg daily.	Awan et al., 2022 [30]
p. (Gly676Alafs*33)	FH (2 families)	Saudi	12	Statin + ezetimibe + evolocumab	Variability in clinical manifestation and resistance to multiple treatment regimens depend on variant zygosity. Patients were managed with LDL-apheresis.	Awan et al., 2021 [34]
p. (Gly676Alafs*33)	Hom-FH	Saudi	2	Rosuvastatin 40 mg, ezetimibe 10 mg, evolocumab 420 mg/month, and lomitapide 5–40 mg	Lomitapide has been shown to significantly lower cholesterol and CVD events.	Mahzari et al., 2021 [53]

**^#^** Statin maximum tolerated dose: simvastatin 80 mg, pravastatin 40 mg, lovastatin 80 mg, fluvastatin 80 mg, atorvastatin 80 mg, or rosuvastatin 20–40 mg. FH, familial hypercholesterolemia; Het-FH, patients with heterozygous FH; Hom-FH, patients with homozygous FH; CVD, cardiovascular diseases.

## Data Availability

The data presented in this study are available on request from the corresponding author. The data are not publicly available due to privacy of the patients.
